# Recreational Use of the Countryside: No Evidence that High Nature Value Enhances a Key Ecosystem Service

**DOI:** 10.1371/journal.pone.0165043

**Published:** 2016-11-09

**Authors:** Karen Hornigold, Iain Lake, Paul Dolman

**Affiliations:** School of Environmental Sciences, University of East Anglia, Norwich, United Kingdom; University of Missouri Columbia, UNITED STATES

## Abstract

In Western Europe, recreational amenity is presented as an important cultural ecosystem service that, along with other values, helps justify policies to conserve biodiversity. However, whether recreational use by the public is enhanced at protected areas designated for nature conservation is unknown. This is the first study to model outdoor recreation at a national scale, examining habitat preferences with statutory designation (Site of Special Scientific Interest) as an indicator of nature conservation importance. Models were based on a massive, three year national household survey providing spatially-referenced recreational visits to the natural environment. Site characteristics including land cover were compared between these observed visit sites (n = 31,502) and randomly chosen control sites (n = 63,000). Recreationists preferred areas of coast, freshwater, broadleaved woodland and higher densities of footpaths and avoided arable, coniferous woodland and lowland heath. Although conservation designation offers similar or greater public access than undesignated areas of the same habitat, statutory designation *decreased* the probability of visitation to coastal and freshwater sites and gave no effect for broadleaved woodland. Thus general recreational use by the public did not represent an important ecosystem service of protected high-nature-value areas, so that intrinsic and existence values remain as the primary justifications for conservation of high nature value areas. Management of ‘green infrastructure’ sites of lower conservation value that offer desirable habitats and enhanced provision of footpaths, could mitigate recreational impacts on nearby valuable conservation areas.

## Introduction

Nature-based recreation and subsequent well-being are presented as an important cultural ecosystem service [1,2] that is increasingly used to support investment in biodiversity conservation [3,4]. Evidence is, however, surprisingly scarce [5]. Interacting with nature benefits physical health (reducing stress levels and mortality), cognitive performance (reducing mental fatigue) and well-being (elevated mood and self-esteem) [5,6]. On a global scale, visits to protected natural areas (PAs) are on the increase [4] and there is evidence that PAs holding greater levels of biodiversity are preferentially visited by nature-based tourists [7,8]. In the UK, statutory conservation policies increasingly emphasise societal benefits of connection to nature [3,9]. A core conservation strategy is the protection of areas that support characteristic and or threatened habitats and species (henceforth ‘high nature value’ areas); however, whether the general public making every day recreational visits place greater value on such high nature value areas designated for their biodiversity, versus the wider countryside, is unknown. This is especially important in Europe where there are many opportunities to pursue recreational activities in other types of ‘green space’.

Recreationists can have undesirable effects on high nature value areas [10,11] that may be mitigated by re-distributing recreational pressure to other areas of lower nature value; yet public access to nature is essential to build a constituency for conservation [12,13] and use gives amenity value with potential to generate conservation revenues [4]. Strategic management of recreational provision would be strengthened by better understanding the importance of high nature value areas relative to the wider countryside. PAs across England deliver biodiversity benefits but fewer recreational visits than predicted from their relative area [14]. However, recreational preferences were not evaluated as analyses did not control for local population density, that was twice as high in the vicinity of visits to the wider countryside than visits to PAs, and also did not control for effects of access networks or preferred land cover types. Sen et al. [15] assessed the economic value of recreation, modelling land cover class, travel distance, socio-demographics and population; but did not examine whether conservation status affected visitation preferences.

Here the ecological preferences underlying recreational behaviour are identified, using a nationwide sample of over 30,000 spatially referenced visits across the countryside and a greater number of randomly selected control sites, to model the influence of land cover on the probability of site visitation. In the densely populated and intensively managed UK countryside and farmland, recreation opportunities are provided by an extensive and continuous footpath network, providing public access and legal rights of way throughout the wider countryside including across privately owned land. We therefore control for access in terms of road proximity for travel to sites, and footpath density facilitating entry into sites, as well as travel cost, source population density and regional behavioural differences. The effect of high nature value on likelihood of site visitation is examined considering as a proxy whether land cover within the site has statutory designation as a Site of Special Scientific Interest (SSSIs). SSSIs represent the UKs most important sites for biodiversity conservation [16], are designated using objective criteria and include all National Nature Reserves (NNRs) and Natura 2000 sites designated under European Directives. The aim is to determine whether recreational use is an important ecosystem service provided by high nature value areas relative to the wider countryside. Irrespective of whether recreation amenity provides additional justification for conservation, understanding which habitats are in greatest demand informs the provision of green infrastructure and recreation opportunities to mitigate recreational pressure on vulnerable conservation areas.

## Methods

### Study design

**Fig 1:**
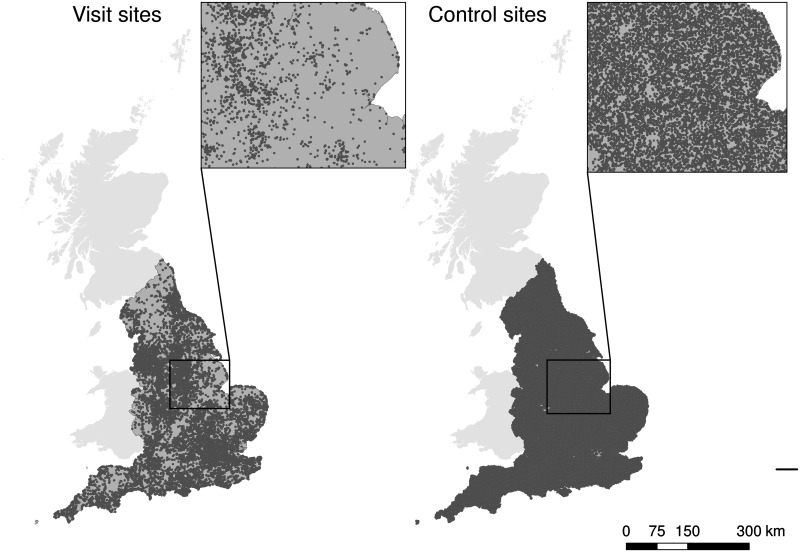
Distribution within England of visit and control points used in this study.

An unpaired case-control design [17] was used to compare characteristics of recreational visit sites with a set of randomly selected available countryside sites (controls) not used by respondents in this study. Point visit locations were taken from the Monitor of Engagement with the Natural Environment (MENE) survey (2009–2012) of recreational activity by English households [18], aggregating data across years. This was collected using a nationally representative sample of face-to-face, in-home interviews, conducted each week of the year. During each interview, one recreational visit undertaken by the respondent in the week preceding the interview was selected randomly and the location recorded as a grid reference (Ordnance Survey National Grid). Details of the systematic sampling protocol that achieved maximum geographical dispersion and a balanced sample of adults in every week of the survey, as well as the full questionnaire, are provided in the MENE technical report [18]. In total, 44,485 visit locations were obtained and mapped as points in ArcGIS 10.1 (ESRI Redlands, USA), representative of recreation activity of the national population throughout the year. In order to examine the extent to which nature-based recreationists preferentially use high nature value sites relative to other sites in the wider countryside, visits in predominantly built-up areas (classed as sites containing > 70% built-up land cover) were first excluded to filter visits to urban or sub-urban parks, recreation grounds, and playgrounds (see S1 Appendix in Supporting Information). After filtering, 31,502 countryside visits remained (hereafter ‘visit points’). Twice as many controls (63,000) were generated (hereafter ‘control points’) using Geospatial Modelling Environment (GME) [19], randomly located within the boundaries of England but constrained to be at least 25m from visit points so that control points could not be placed in a known visit location. This control design was chosen as recreational access to lands in the UK is not limited to designated recreational areas; by law every person has the right to walk, ride and cycle on a dense network of public rights of way throughout the countryside including privately owned land. Therefore both visit and control sites in this study represent recreational opportunities in a broad range of public and private lands, whereby access is largely determined by road and path networks. Controls within predominantly built-up areas were excluded in the same way as for visits (Fig 1). A quasi-experimental design was tested also, with control points stratified by distance-weighted population, a combined measure of travel cost and population density surrounding visit points (see below); but random controls were considered superior as an explicit measure of population and travel cost could be included in models (see S1 Appendix). Recreationists generally visit an area (e.g. to walk or cycle) not just a point location (i.e. as obtained from the MENE survey), but spatially-explicit information on detailed movements and routes taken during the visit were not available. Therefore, visit and control points were buffered by a 400m radius to represent the area visited, as empirical studies of visitor countryside access patterns show that this is a typical recreational penetration distance (see S1 Appendix). Nevertheless, sampling land cover from within such buffers will introduce some unavoidable error. Buffered visit and control points are hereafter referred to as ‘visit sites’ and ‘control sites’, or jointly as ‘sites’. Due to the heterogeneous nature of land cover within each visit site (i.e. unique mix and relative proportions of land covers within each site), statistical matching to pair conservation designated sites with non-designated sites that have similar characteristics was infeasible. To account for potential alternative or substitute sites that may affect visitation rates to the focal site, land cover composition was examined within a 10km radius buffer around sites (considered appropriate as 82% of respondents reported travelling less than 5-8km), hereafter referred to as the ‘surrounding landscape’.

### Predictors of visitation

In order to obtain the proportion of land covers within visit and control sites (and the surrounding landscape), the 25m resolution Land Cover Map 2007 (LCM2007) [20] was used. The 22 LCM2007 land cover classes were aggregated into 11 broad classes to obtain robust data in terms of reliability of classification and sufficient sample size; some land cover classes in LCM2007 were not distinguished reliably using spectral signature (e.g. neutral, calcareous, acid and rough grassland [20]) which were therefore grouped as semi-natural grassland, and others were limited in area (e.g. supra-littoral rock and littoral rock which were therefore also grouped). Visit and control sites were overlaid on the aggregated land cover map and the proportionate land cover within sites extracted. Land cover classes appearing in fewer than 10% of visit sites were excluded from analysis due to insufficient power (following Boughey et al. [21]), so that 9 classes remained (Table 1). Within each site (and surrounding landscape) land covers were further divided by designation as SSSI (e.g. proportion of buffer supporting broadleaf woodland SSSI, proportion supporting broadleaf woodland non-SSSI) using SSSI boundaries from Natural England [22], with the exception of built-up land and improved grassland that are not statutorily designated as SSSIs and arable, which is rarely designated. SSSIs cover more than 8% of the country with the majority (98% of total area) designated for biodiversity (e.g. richness, representativeness) and or nature conservation (e.g. species of national or international conservation concern) [23]. Non-SSSI land may be public or private land that is either not designated for conservation or recreation, or has some other designation but is not a SSSI.

**Table 1:** Candidate variables used to model likelihood of site visitation by recreationists.

Code	Predictor	Units	Description
Comp	Arable^a^	Proportion within site or surrounding landscape	Proportion of annual and perennial crops and freshly ploughed land
	Coast^a^	Proportion within site or surrounding landscape	Proportion of sand dunes, shingle, littoral mud and littoral sand
	Broadleaved woodland^a^	Proportion within site or surrounding landscape	Proportion of broadleaved woodland with >20% tree cover or >30% scrub cover
	Built-up^a^	Proportion within site or surrounding landscape	Proportion of urban and suburban areas including towns, cities (and residential gardens), car parks and industrial estates
	Coniferous woodland^a^	Proportion within site or surrounding landscape	Proportion of coniferous woodland with >20% cover
	Freshwater^a^	Proportion within site or surrounding landscape	Proportion of lakes, canals, rivers and streams
	Improved grassland^a^	Proportion within site or surrounding landscape	Proportion of grassland modified by fertiliser and reseeding typically managed as pasture or mown
	Lowland heath^a^	Proportion within site or surrounding landscape	Proportion of heather and dwarf shrub, gorse and dry heath below 300m a.s.l. as defined by Gimingham [24], delimited according to the digital terrain model OS Terrain 50 [25]
	Semi-natural grassland^a^	Proportion within site or surrounding landscape	Proportion of neutral, calcareous, acid and rough grassland
Pop	Weight.pop.2^b^	No. people	Total number of people residing within 10km of the site, inverse-weighted by distance squared from visit and control points
Cty	County^c^	85 levels	County in which the site is located
Path	Path.length^d^	m	Total length of path network within site
Elev	Mean.elev^e^	m	Mean of all Digital Terrain Model 50m cells within site
Road	Dist.Aroad^f^	m	Distance from visit and control points to nearest major road

^a^LCM2007

^b^km resolution population raster created from 2011 ONS census data

^c^Assigned according to county boundaries downloaded from http://www.gadm.org/

^d^OpenStreetMap

^e^OS Terrain 50

^f^OS Meridian

The larger the resident population near a site, the more likely it is to be visited, with source population effects diminishing with distance due to increasing travel cost (and time). To account for this, a measure of inverse distance-weighted population around sites was included in models. Population data from the 2011 census of households provided by the Office for National Statistics [26] (England and Wales) and National Records of Scotland [27] were linked to coordinates, using the UK Postcode Directory [28] and aggregated into 1km cells to create a UK-wide population raster (some visits close to the borders may originate from Wales or Scotland). This was used to extract gridded population data within a 10km radius of site centroids. Three population distance-density functions were then tested, of which weight.pop.2 (population weighted by inverse of distance squared) best distinguished visit from control sites in a univariate GLM (see S1 Appendix for further details) and was included in all subsequent models.

Outdoor recreation in the UK is enabled by a network of public rights of way. In the UK there is no central digital repository for such data hence a path network layer encompassing bridleways, cycleways, footpaths, paths and tracks was extracted from OpenStreetMap [29]. These data, collected by contributors using GPS devices, aerial imagery and field maps, were extensively validated against rights of way shown on OS raster maps (see S1 Appendix) and found to be a good representation of public rights of way. Access and recreational opportunity within sites was indexed as the path network length within sites, while access to arrive at sites was indexed as the straight line distance from visit or control points to the nearest major road (A Road) [30].

Mean elevation of sites (extracted from OST50 [25]) was selected *a priori* as a predictor variable. Sites with lower mean elevation were expected to have a greater probability of visitation, as those engaging in arduous activity are a subset of recreationists. Elevation gain within sites was explored but provided less explanatory power.

As recreational visits to SSSIs and non-SSSIs were being compared, potential access constraints between these were examined. Models control for access to sites via the road network and entry within sites via the public footpath network, however on much private land (including privately-owned SSSIs) recreationists are constrained to walk along footpaths without entry being permitted to the adjacent land. In contrast, areas either having a statutory right of access under the Countryside Rights of Way Act (CRoW), or as Country Parks (CPs) or Local Nature Reserves (LNRs), that together cover 8.5% of England, allow visitors the ‘freedom to roam’. These ‘open access’ areas were mapped and the proportion cover compared between SSSI and non-SSSI land while controlling for land cover type, to examine any potential bias. In a further subsidiary analysis land cover classes were divided by designation as National Nature Reserves (NNRs) as a proxy for high nature value sites with public access. NNRs are high-quality SSSIs used to showcase conservation management and engage the public and thus are areas of high nature value where recreational access is encouraged. Models using NNRs are compared with those using SSSI designation.

### Analysis

Generalised linear mixed models (GLMMs) with binomial error and logit link function predicted *P*(Visit_i_), the probability of a recreational visit to site *i*, as a function of the proportions of site land cover classes (Comp_*i*_), mean elevation, distance from nearest major road and path density (fixed effects, Table 1), controlling for distance-weighted population and county (random effects). Counties (categorical, 85 levels, from database of Global Administrative Areas [31]) vary in area from 28km^2^ to 7965km^2^ with a mean population of 644,944. The interaction between weight.pop.2 and county allowed for potential differences in *per capita* frequency of recreational activity due to socioeconomic or cultural effects. Predictor variables were centred and scaled with zero mean and unit standard deviation for comparability of coefficients [32]. Three GLMMs were fitted; in model 1 all site land cover classes were included once (eq 1), in model 2 variables for the proportions of land covers within the surrounding landscape were also included (Lands_*i*_; eq 2), in model 3 selected site land cover classes were divided into areas designated and non-designated as SSSIs and landscape land cover variables (Lands_*i*_) were excluded (eq 3):

$$P\left(Visit_{i}\right)=f\left(Comp_{i},Elev_{i},Road_{i},Path_{i},Pop_{i},Cty_{i}\right)$$ (1)

$$P\left(Visit_{i}\right)=f\left(Comp_{i},Elev_{i},Road_{i},Path_{i},Pop_{i},Cty_{i},Lands_{i}\right)$$ (2)

$$P\left(Visit_{i}\right)=f\left(Comp.non-des_{i},Comp.des_{i},Elev_{i},Road_{i},Path_{i},Pop_{i},Cty_{i}\right)$$ (3)

Differences between equivalent non-designated and designated (model 3) land cover coefficients, were evaluated by Z tests.

GLMMs were fitted using the lme4 package [33]. Inspection of correlograms established that spatial autocorrelation was negligible in both models (see S1 Appendix). Model fit was evaluated using a pseudo *R*^2^ specifically developed for GLMMs, which gives an estimate of the variance explained by fixed effects (marginal *R*^2^) and both fixed and random effects combined (conditional *R*^2^) [34]. Predictive performance of the two m odels was evaluated against independent data from the subsequent 2012–2013 MENE survey (n = 10,622) and additional random controls (n = 10,622). For each model, AUC—the area under the receiver operating characteristic (ROC) curve—was calculated using the pROC package in R [35]; AUC ranges from 0.5 for models that perform no better than random, to 1 for models with perfect discrimination [36]. Whether AUC values (and thus model prediction accuracy) differed significantly among models was tested (following DeLong et al. [37]) within the pROC package.

## Results

### Recreationists’ preferences for site characteristics

Examining effects of site characteristics upon visitation probability without considering designation status indicated a strong positive influence of path density (mean within visit sites 2055m ± 1916 SD; within control sites 604m ± 865 SD, Table 2). Visitation probability was strongly reduced for sites at higher elevation or far from a major road. Intercepts for each county ranged from -1.45 ± 0.16 95% CI to 1.14 ± 0.24 95% CI and weight.pop.2 coefficients from -1.67 ± 0.95 95% CI to 2.26 ± 0.61 95% CI (S1 Fig), showing variation in *per capita* visitation probability between counties and supporting inclusion of these random effects.

**Table 2:** Generalised linear mixed model predicting recreational demand in the countryside, controlling for population and county.

	Standardised Coefficient	Std. Error	z	P
*Non-land cover variables*				
Path length (access within site)	0.826	0.014	59.96	***
Elevation	-0.370	0.017	-22.22	***
Distance to major road (access to site)	-0.132	0.013	-9.83	***
*Land cover classes with positive effect*				
Built-up	0.631	0.022	29.14	***
Coast	0.287	0.016	18.49	***
Freshwater	0.161	0.010	16.26	***
Broadleaved woodland	0.158	0.015	10.37	***
*Land cover classes with negative effect*				
Arable	-0.645	0.031	-20.70	***
Improved grassland	-0.129	0.022	-5.80	***
Lowland heath	-0.080	0.012	-6.64	***
Coniferous woodland	-0.078	0.013	-6.18	***
Semi-natural grassland	-0.043	0.016	-2.73	**
*Constant*	-0.697	0.077	-9.06	***

Dependent variable: the likelihood of visitation. *P*<0.001 ‘***’, *P<*0.01 ‘**’

As predictor variables were standardised, the relative size of coefficients indexes the relative magnitude of response to a one SD increase in the magnitude of the predictor. Of the semi-natural land cover classes, coast had the strongest positive effect on the probability of visitation, followed by freshwater and broadleaved woodland (Table 2). Arable land cover had the strongest negative effect on the probability of visitation followed by improved grassland, lowland heath, coniferous woodland and semi-natural grassland.

**Fig 2:**
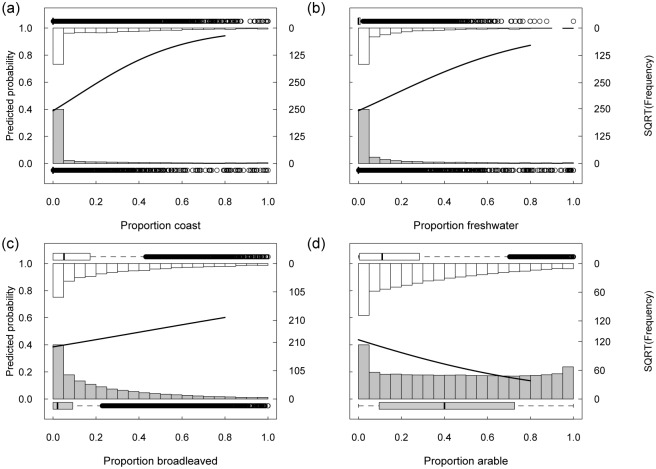
Predicted influence on visitation probability of coast, freshwater, broadleaved woodland and arable. From model 1 (eq 1) controlling for path length, elevation, distance to nearest major road, distance-weighted population and county. Bars show the frequency distribution (square root scaled) within visit (unfilled) and control (grey) sites. Predictions were obtained by varying the proportionate cover of the land cover class shown between 0-0.8. All other land cover classes were held proportional to their mean such that they sum to 0.2 (so that total land cover proportion did not exceed 1). Control variables were held at their mean. Horizontal box and whisker plots show median, quartiles and outliers of land cover proportions in visit (unfilled) and control (grey) sites.

To visualise how the probability of visitation (positive or negative) responds to changes in land cover proportion, Fig 2 was produced for the four land covers with the strongest influence. The probability of visitation was 50% at proportionate covers of coast and freshwater of 0.11 and 0.15 respectively (Fig 2a & 2b), whereas a greater cover of broadleaved woodland (approximately 0.43 and above; Fig 2c) was required before a site was more likely to be visited than not. Arable had the strongest negative effect on visitation probability, with a large effect size relative to other land cover classes (Fig 2d). Recreationists were less likely to visit sites comprising a greater proportion of lowland heath, improved and semi-natural grassland or coniferous woodland. Addition of land covers in the surrounding landscape to the model made negligible difference to within-site land cover coefficients and did not alter the significance direction or relative strength of effects (S3 Fig). Therefore the surrounding landscape is omitted in further modelling that compared effects of SSSI versus non-SSSI land cover.

### Effect of conservation designation

Preferences for land cover classes of potential conservation importance were then examined separately, according to whether they were SSSI designated. For the three land covers with the strongest positive effects on visitation probability and the land cover with the strongest negative effect, 6–20 times as much of the designated SSSI area permitted ‘open access’ (the right to roam freely across land, not constrained to a recognised footpath, combining CROW, CP, LNR) (coast, 6.2%; freshwater, 17.1%; broadleaved woodland, 29.7%; lowland heath, 86.0%) than for equivalent land cover not designated as SSSI (open access: 0.3%, 1.4%, 5.2% and 4.1% respectively). Therefore, all else being equal, a greater visitation rate may be expected to habitat designated as SSSI.

**Fig 3:**
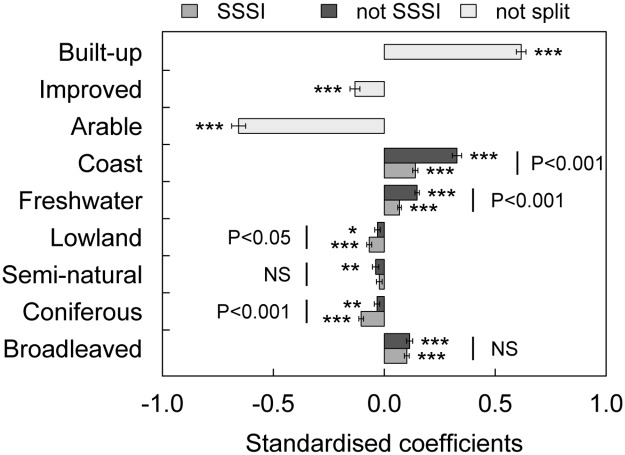
Effects on visitation probability of non-SSSI-designated or SSSI-designated land covers. Standardised coefficients from model 2 (eq 3) controlling for path length, elevation, distance to nearest major road, distance-weighted population and county. Bars denote standard error. For each land cover, *P* values of *Z*-tests compare pairs of coefficients between non-SSSI-designated/SSSI-designated (*P*<0.001 ‘***’, *P*<0.01 ‘**’, *P*<0.05 ‘*’).

Splitting land covers by SSSI designation increased model support (Δ AIC = -186), but with only a slight increase in predictive ability (Model 1: AUC = 0.8425 ± 0.005 95% CI; Model 2: AUC = 0.8430 ± 0.005 95% CI; *Z* = 2.44, *P* < 0.05) and model fit (marginal *R*^2^ for model 2 was 0.44 compared to 0.43 for model 1). The appeal of broadleaved woodland was similar irrespective of whether it was designated an SSSI (*Z =* 0.7, *P* = 0.47; Fig 3) with little difference between coefficients (Δ = -0.013 ± 0.018 SE), whereas the attractiveness of coast and freshwater was significantly greater when not designated (Fig 3). While non-designated coast and freshwater coefficients were close to the original (model 1) coefficient error bounds, SSSI-designated coefficients were lower (designated versus non-designated: coast Δ = -0.188 ± 0.024 SE, *Z =* 7.8, *P* < 0.001; freshwater Δ = -0.079 ± 0.013 SE, *Z =* 6.2, *P* < 0.001). Effects of designating the freshwater or coastal area within a site was examined separately for low (20%) and high (80%) overall cover, holding remaining land cover classes constant in proportion to their national mean. Freshwater designation minimally affected visitation probability at low cover (0.559 non-designated, 0.518 designated) but at high cover visitation probability was lower with designation (0.900 non-designated, 0.813 designated; S2 Fig). Coastal visitation probability was substantially lower with designation (low cover: 0.748 non-designated, 0.544 designated; high cover: 0.996 non-designated, 0.865 designated; S2 Fig). The negative effect of semi-natural grassland did not differ with designation, whereas for coniferous woodland and lowland heath visitation probability was significantly lower when designated (*Z =* 4.45, *P* < 0.001 and *Z =* 2.20, *P* < 0.05 respectively). Despite the encouragement of access within NNRs, subsidiary analysis contrasting land covers designated or non-designated as NNRs was consistent with SSSI results for broadleaf woodland, coast, freshwater and semi-natural grassland, but lowland heath and coniferous woodland NNR-designation had no significant effect on visitation probability (S4 Fig).

## Discussion

For preferred land cover classes there was no evidence that high nature value areas had greater appeal despite having greater levels of permitted open access. Recreation was previously found to be under-represented by protected areas in England, but in analyses that did not control for source population density, road access or footpath density [14]. Controlling for these factors, this study provides clear evidence that high nature value (inferred by statutory designation as SSSI) does not confer additional recreational value for the general public. This has important implications for justifications of biodiversity conservation.

When a land cover was of elevated conservation importance recreational use by the wider public was not enhanced and in the case of coasts and freshwater it was less likely to be visited. Thus while the public sought access to the countryside or greenspace, this was independent of the nature conservation quality of these locations. Dallimer et al. [38] found no consistent relationship between species richness and human well-being in a survey of visitors to riparian greenspaces, but a positive effect of *perceived* richness. Conservation importance may not strengthen the broader cultural service of recreational opportunities obtained from ecosystems if this is not recognised or sought by most recreationists or the general public. Whilst biodiversity is an important factor for nature tourists visiting national parks in Finland [8] and protected areas in Uganda [7] this is based on a self-selected sample of nature enthusiasts. Similarly, nature-watching is a popular recreation in the UK [39]. However there was no evidence that high nature value plays a role in recreational site selection for day-to-day use based on a representative nationwide sample of the general public. As SSSI designation did not add to the appeal of sites for most recreationists, the public expenditure on these highly valued conservation areas (£85.4 million in England in 2008–09) [40] whilst underpinning biodiversity conservation does not bring benefits in terms of recreational amenity of the general public. Most public benefits are likely expressed through non-use values [41].

Accepting the importance and necessity of conservation areas, pressure on vulnerable sites may be mitigated by providing recreational opportunities in low nature value sites of preferred habitat types. There was a distinct preference for broadleaved over coniferous woodland, a distinction not made in previous studies of forest recreation in Britain [42,43]. Recreation value of coniferous woodlands may therefore be enhanced by planting or retaining broadleaved species along paths. Although broadleaved woodland had clear appeal to recreationists, some other land covers of conservation importance were not preferentially selected. Lowland heaths support species and habitats of European conservation importance that are sensitive to recreational impacts; consequently there has been much research on visitation patterns within heathland [44,45]. Nevertheless on a national level lowland heath was not favoured by recreationists; it may be therefore that lowland heaths are visited when local to recreationists although more desirable land covers remain preferred.

Analyses presented in this paper benefitted from a massive three year sample of spatially-referenced recreational visits to the natural environment collected in a systematic way from a nationally representative sample of the population. Despite these advantages, one limitation was that although the visit location was known, the precise movements within the visited site was not, thus the area used had to be approximated using a 400m buffer. Furthermore, the scale of our analysis meant that for visit and control sites, detailed information that may influence visitation (e.g. car parks, facilities) could not be obtained in a comprehensive manner. However, we argue that the most important elements likely to affect probability of visitation such as land cover, access to site and access within site were all accounted for in our models. Lastly, while the survey design provided a representative sample of week-to-week recreational behaviours throughout the year, less frequent recreational visits (potentially involving longer travel distance to honey pot sites) may be under-represented and require further investigation.

## Conclusions

Understanding the mechanisms driving countryside recreationists’ choice of visit location supports management of the countryside for both recreation and conservation. The relationships derived from a nationally representative sample of English households are likely to be relevant to other developed, urban based countries. Further studies are required however to gain a better understanding of cultural differences in the importance of nature value for general recreation, as the global picture may highlight differing trends as with nature-based tourism [4]. This study shows that, in spite of enhanced well-being from contact with nature being frequently presented as an important ecosystem service and used to support investment in conservation, there is no ecosystem service gain from higher nature value in terms of recreational value to the general public. Protected areas benefit the wider public through non-use values and reconciliation of conservation and recreation remains pertinent.

## Supporting Information

S1 Appendix Additional methodological details. (DOCX)

S1 Fig Point estimates and 95% confidence intervals of random effect parameters. (PDF)

S2 Fig Predicted influence on visitation probability of freshwater and coast when SSSI designated and non-designated. (PDF)

S3 Fig Comparison of the effects of within-site land covers on visitation probability from a model without surrounding landscape variables (model 1) and a model with landscape variables included (model 2). See Table 1 for definition of variables. Bars denote standard error. *P*<0.001 ‘***’, *P*<0.01 ‘**’, *P*<0.05 ‘*’. *Z*-tests comparing pairs of coefficients of the same land cover type were non-significant (NS) as shown next to each pair.
(PDF)

S4 Fig Effects on visitation probability of non-NNR-designated or NNR-designated land covers. Standardised coefficients from a GLMM controlling for path length, elevation, distance to nearest major road, distance- weighted population and county. Bars denote standard error. For each land cover, *P* values of *Z*-tests compare pairs of coefficients between non-NNR-designated/NNR-designated (*P*<0.001 ‘***’, *P*<0.01 ‘**’, *P*<0.05 ‘*’).
(PDF)
