# Supplementary material for: Recreational Use of the Countryside: No Evidence that High Nature Value Enhances a Key Ecosystem Service
Source: PLoS One. 2016 Nov 9;11(11):e0165043. doi: 10.1371/journal.pone.0165043 (PMC5102377; doi:10.1371/journal.pone.0165043)
Supplement: S1 Appendix — (DOCX) [file pone.0165043.s001.docx]

**Appendix S1.**Additional methodological details

*Methodology for defining ‘countryside’ visits i.e. exclusion of visits to urban green spaces*

As the focus of this study is to examine the effects of outdoor recreation on semi-natural habitats, we excluded visits in built-up areas. The dataset included all recreational visits to the ‘natural environment’ (including for example urban recreation grounds, parks and playgrounds), only excluding shopping trips and visiting allotments or personal gardens. To selectively remove ‘urban green spaces’ the proportion of built-up area (extracted from Land Cover Map 2007 [1]) within buffers surrounding visit points was examined and a cut-off point applied to define a visit location as built-up or countryside. Cut-off points of 50%, 60%, 70%, 80% and 90% built-up area were examined at buffer sizes of 200, 400, 600, 800m radii (see Table A).

The visit localities that were retained were examined spatially overlaid on the land cover map (LCM2007) for East Anglia to see where these were in relation to built-up areas. Visit localities retained under each percentage cut-off at each buffer radius in Table A were examined in turn and it was determined that the 70% cut-off within 400m buffers performed best (highlighted in bold) as visit localities close to large areas of open space within built-up areas and on the edge of cities were retained, whilst visit localities within large homogenous built-up areas were removed. At the 70% cut-off approximately 25% of observed visits were excluded due to being within built-up areas (Table A).

**Table A.** Number of visits excluded from observed visit (MENE) dataset (n = 44,495) at different exclusion thresholds for built-up area within buffers and at different buffer radii

| **% built-up area** | **Buffer radius** | | | |
| --- | --- | --- | --- | --- |
|  | **200m** | **400m** | **600m** | **800m** |
| >50 | 19303 | 19617 | 19146 | 18444 |
| >60 | 15968 | 15776 | 15225 | 14565 |
| >70 | 12851 | **12301** | 11796 | 10903 |
| >80 | 9848 | 8579 | 7769 | 7049 |
| >90 | 6721 | 4730 | 3704 | 2990 |

*Methodology for creation of stratified controls, analysis using stratified control sample and comparison of results from models with stratified controls and random controls*

We tried an alternative method of controlling for the effects of local source population density and travel cost. Instead of including distance-weighted source population (weight.pop) as a random effect in the model, we stratified the controls (i.e. used a new set of controls generated by stratified random sampling) to have a similar frequency histogram of distance-weighted source population as visit localities; thus we controlled for population and travel distance effects through locating controls in areas with a similar source population within the 10km travel distance as the visit sample, making the weight.pop variable redundant. By allocating a similar ratio of control to visit localities within intervals of distance weighed population (see below) this allowed conclusions to be made regarding the relative of pull of different land cover types without confounding effects of local population density or proximity to residential areas.

The stratified controls were generated as follows. The frequency histogram of distance-weighted population for visit localities gave the number that fell within 22 bands of 5000 increments between the minimum weight.pop (zero) and maximum (110,000). The counts were then doubled to get the sample sizes for controls within the same bands. To generate control points, random points were placed within the boundaries of England and weight.pop was calculated until the sample sizes for each weight.pop band was achieved. These points were then buffered by 400m to create the final control localities. Model 2 (see section 2.3 in main paper) was run using the existing visit localities and the new stratified control localities and resulting coefficients were compared to original model coefficients (Fig A). The effect of significantly positive land covers were very similar to the original model. Non-significant and significantly negative land cover coefficients show some differences but their effect sizes remain trivial.


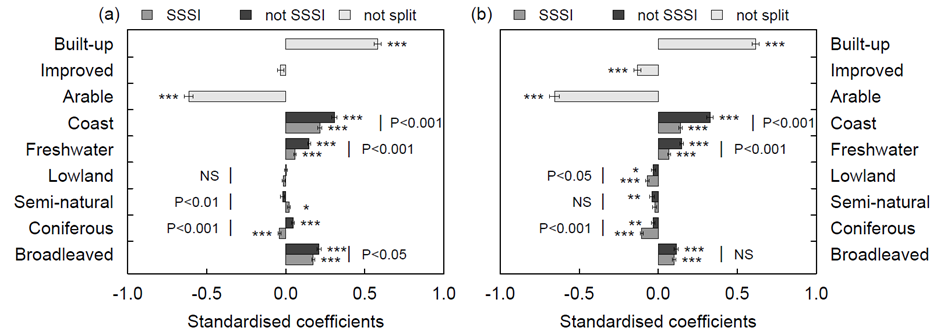


**Fig A.** Effects on visitation probability of land covers separately outside or inside an SSSI, showing standardised coefficients from (a) stratified control model (b) random control models (controlling for path length, elevation, distance to nearest major road, weighted population and county in both); bars denote standard error. *P*<0.001 ‘***’, *P*<0.01 ‘**’, *P*<0.05 ‘*’. *Z*-tests comparing pairs of coefficients of the same land cover type are shown next to each pair (NS denotes non-significant)

*Justification for choice of buffer radius within which to extract characteristics of the area surrounding visit and control points*

Mean ‘penetration distances’, defined as the ‘linear distance from the mid-point of the route to the access point’ [2] were 777m for dog walkers (n=3), and 795m for walkers (n=2), for three heathland complexes in southern England. As these are large protected areas (mean size 5421 ha, range 2742-8400 ha), they are not likely to be representative of the visit behaviour captured in this study as the majority of visits will take place locally (over 80% of respondents reported travelling less than 5 miles to their destination). Mean penetration distance reduces consistently with a reduction in site area [3] which lends support for a smaller buffer size for this study. Hence a buffer of 400m radius was chosen with which to extract characteristics of the area visited. Buffering the point representing visit locations will also account for any uncertainty in the initial mapping by surveyors, as although precise (1m) grid references were provided, often respondents could only give a description of where they went.

*Validation of path network layer*

For validation of the path network layer [4], 1km grid squares were randomly placed in East Anglia (n=100) and the North East (n=100) and the OpenStreetMap path network was visually compared to paths mapped by Ordnance Survey (OS) VectorMap^®^ (definitive but not available in a useable digital format) within each grid square. In East Anglia 87% of grid squares had fully corresponding paths and in the North East 72%, thus it was concluded that the path network layer was a good representation of reality.

*Determining best fitting distance-weighted population function*

To obtain inverse distance-weighted population per locality, 1km resolution Euclidean distance rasters with a 10km radius around visit and control points were generated and multiplied with the population raster as per eqn 1-3. A 10km radius was chosen as 82% of respondents reported travelling less than 5-8km. Three different distance weightings were applied to determine the best function for these data (equations 1-3):

weight.pop.1 = pop * (1/d) AIC = 104,393 (eqn 1)

weight.pop.2 = pop * (1/d^2^) Δ AIC from eqn 1 = -4,973.9 (eqn 2)

weight.pop.3 = pop * (1/d^2.5^) Δ AIC from eqn 1 = 9,760.5 (eqn 3)

Where pop = number of people per km^2^

d = distance from focal 1km x 1km cell that the visit or control point lies within, calculated as d = (centroid distance from focal cell centroid (m) + 1000)/1000 so that the maximum weighting of 1 was assigned to the focal cell

To determine the best fitting function these three distance-weighted population metrics were entered separately into univariate generalised linear models (GLMs) with logit link and binomial error structure, to estimate visitation probability (using visit and control localities as a binomial response variable) as a function of surrounding distance-weighted population. Weight.pop.2 provided the best fit to the data based on AIC and thus was included in all subsequent multivariate models.

To check if built-up area shared some explanatory power with weight.pop.2, the proportion of built-up area within localities (i.e. 400m buffers) was added to a model with only weight.pop.2. Upon addition, the weight.pop.2 coefficient was reduced from 1.97 ± 0.02 SE to 0.99 ± 0.02 SE, suggesting built-up area partly acted as a proxy for source population. However, they were only moderately correlated (*r* = 0.48, df = 94500, *P* < 0.001) and thus built-up was retained in subsequent models.

*Checking for spatial autocorrelation*

Correlograms of the spatial dependence between residuals from GLMMs (as estimated by Moran’s I) were examined to check for spatial autocorrelation. Correlograms were plotted using the ncf package in R [5] (Fig B). Due to computational limitations, random samples of 10% of the data were used to generate correlograms (repeated three times per model for robustness). Inspection of correlograms led us to conclude that spatial autocorrelation was negligible and supported the use of non-spatial GLMMs.


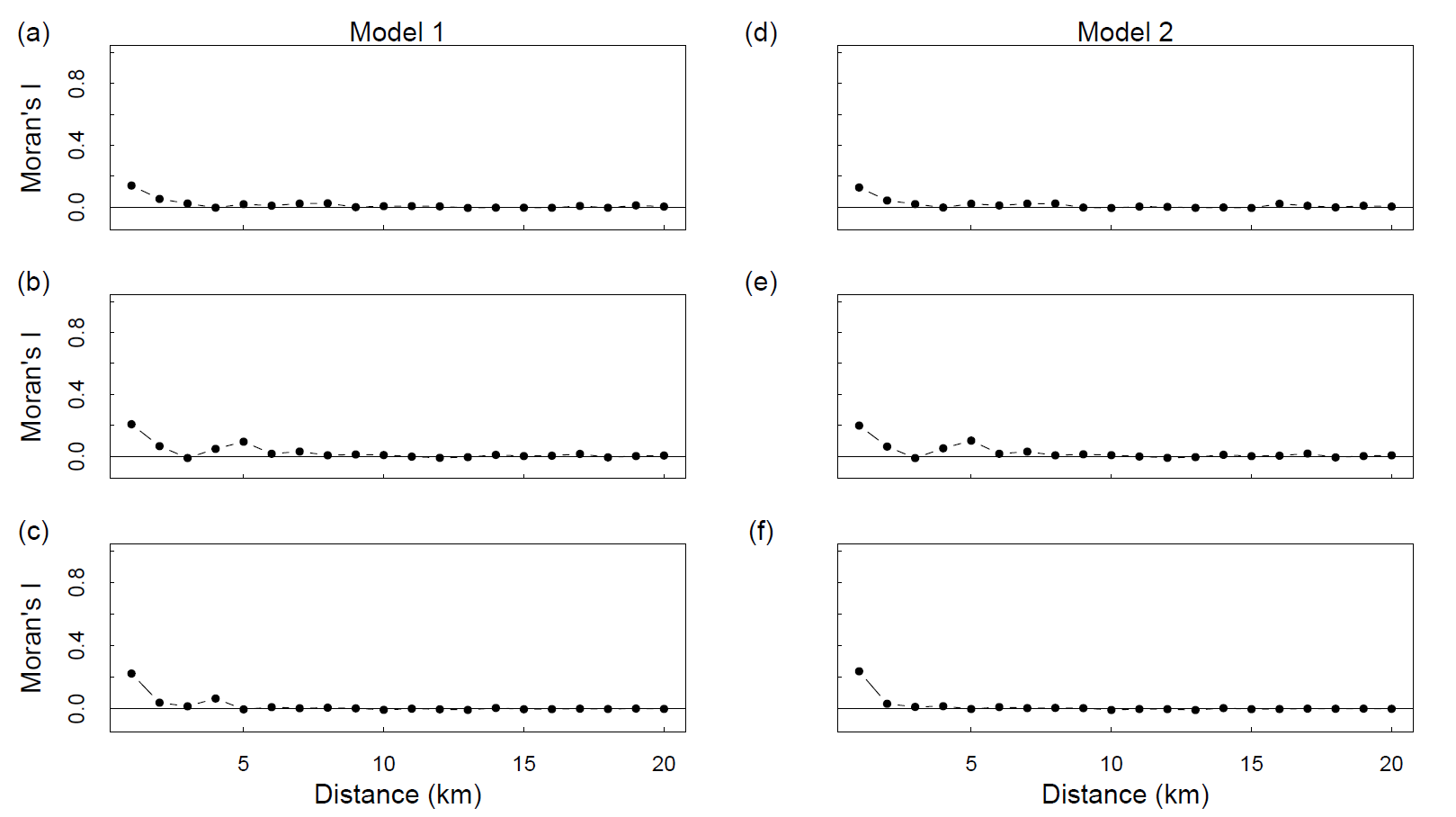


**Fig B.** Correlograms from residuals from (a-c) model 1 which doesn’t consider designation status and (d-f) model 2 where land cover classes are divided into SSSI and non-SSSI (created using three random samples of 10% of the data i.e. 9465 observations due to computational intensity)

**References**

1. Morton D, Rowland C, Wood C, Meek L, Marston C, Smith G, et al. Final report for LCM2007 – the new UK land cover map. Countryside Survey Technical Report No. 11/07 NERC/Centre for Ecology & Hydrology; 2011.

2. Liley D, Jackson D, Underhill-Day JC. Visitor access patterns on the Thames Basin Heaths. Peterborough: English Nature; 2005.

3. Clarke RT, Liley D, Underhill-Day JC, Rose R. Visitor access patterns on the Dorset heathlands. Peterborough: English Nature; 2006.

4. OpenStreetMap. © OpenStreetMap contributors, licensed under the Open Data Commons Open Database License and the Creative Commons Attribution-ShareAlike 2.0 license. [Internet]. Available: http://download.geofabrik.de/europe/great-britain.html.

5. Bjornstad ON. ncf: spatial nonparametric covariance functions. R package version 1.1-5. http://CRAN.R-project.org/package=ncf. 2013.
