# Supplementary figures and images for: Recreational Use of the Countryside: No Evidence that High Nature Value Enhances a Key Ecosystem Service
Source: PLoS One. 2016 Nov 9;11(11):e0165043. doi: 10.1371/journal.pone.0165043 (PMC5102377; doi:10.1371/journal.pone.0165043)

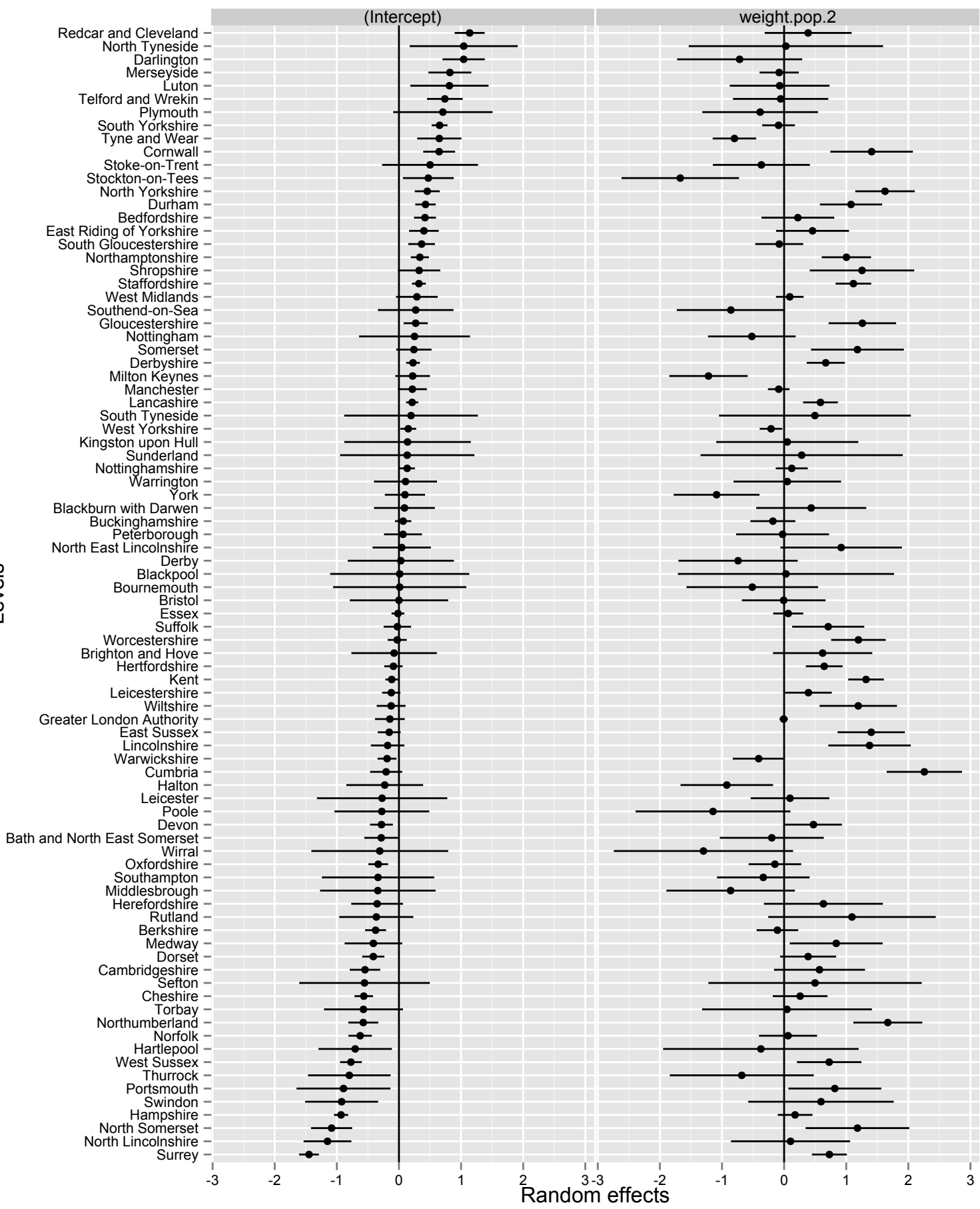

Supplement: S1 Fig — (PDF) [file pone.0165043.s002.pdf]

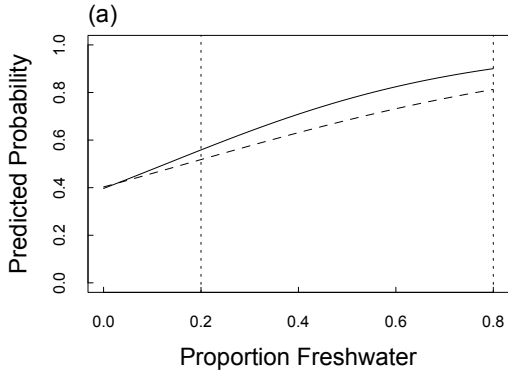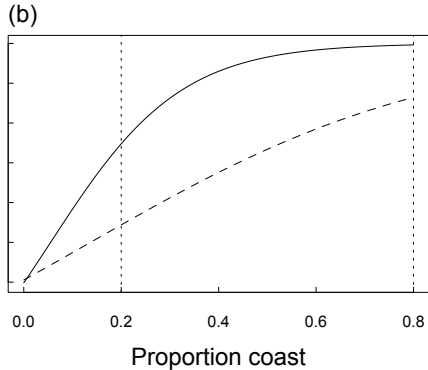

Supplement: S2 Fig — (PDF) [file pone.0165043.s003.pdf]

□ without landscape    ■ with landscape

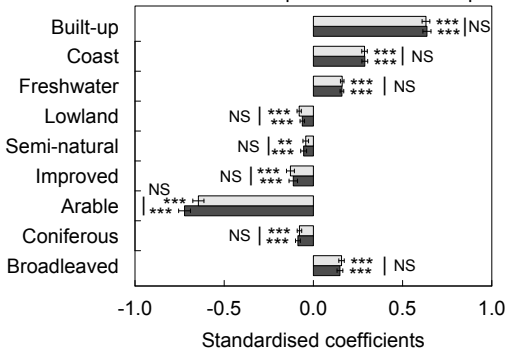

Supplement: S3 Fig — See Table 1 for definition of variables. Bars denote standard error. P<0.001 ‘***’, P<0.01 ‘**’, P<0.05 ‘*’. Z-tests comparing pairs of coefficients of the same land cover type were non-significant (NS) as shown next to each pair. (PDF) [file pone.0165043.s004.pdf]

■ NNR    ■ not NNR    □ undivided

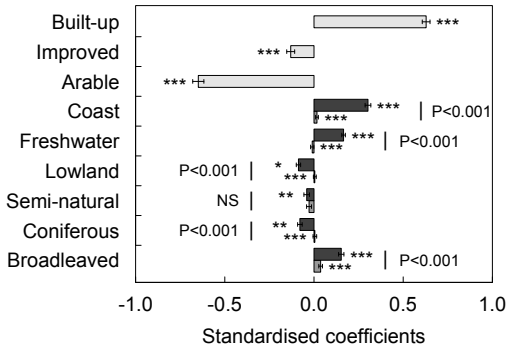

Supplement: S4 Fig — Standardised coefficients from a GLMM controlling for path length, elevation, distance to nearest major road, distance- weighted population and county. Bars denote standard error. For each land cover, P values of Z-tests compare pairs of coefficients between non-NNR-designated/NNR-designated (P<0.001 ‘***’, P<0.01 ‘**’, P<0.05 ‘*’). (PDF) [file pone.0165043.s005.pdf]
